# Patient‐reported symptoms and experience following Guillain‐Barré syndrome and related conditions: Questionnaire development and validation

**DOI:** 10.1111/hex.13367

**Published:** 2021-10-01

**Authors:** Aloysius Niroshan Siriwardena, Joseph N. A. Akanuwe, Vanessa Botan, Despina Laparidou, Ffion Curtis, Jennifer Jackson, Zahid B. Asghar, Timothy L. Hodgson

**Affiliations:** ^1^ Community and Health Research Unit (CaHRU), School of Health and Social Care University of Lincoln Lincoln UK; ^2^ Centre for Ethnic Health Research, Leicester Diabetes Centre University of Leicester Leicester UK; ^3^ Community Orgnisation Group, Lincoln International Business School University of Lincoln Lincoln UK; ^4^ Perception Action Cognition, School of Psychology University of Lincoln Lincoln UK

**Keywords:** experiences, Guillain‐Barré syndrome, patients, patient‐reported experience measure, recovery

## Abstract

**Background:**

Guillain‐Barré syndrome (GBS) is a rare inflammatory peripheral nerve disorder with variable recovery. Evidence is lacking on experiences of people with GBS and measurement of these experiences.

**Objective:**

We aimed to develop and validate an instrument to measure experiences of people with GBS.

**Design:**

We used a cross‐sectional design and online self‐administered questionnaire survey. Question domains, based on a previous systematic review and qualitative study, covered experiences of GBS, symptom severity at each stage, healthcare and factors supporting or hindering recovery. Descriptive, exploratory factor and reliability analyses and multivariable regression analysis were used to investigate the relationships between variables of interest, explore questionnaire reliability and validity and identify factors predicting recovery.

**Setting and Participants:**

People with a previous diagnosis of GBS were recruited through a social media advert.

**Results:**

A total of 291 responders, of different sexes, and marital statuses, were included, with most diagnosed between 2015 and 2019. Factor analysis showed four scales: *symptoms*, i*nformation provided*, f*actors affecting recovery* and c*are received*. Positive social interactions, physical activity including physiotherapy and movement, changes made at home and immunoglobulin treatment were important for recovery. Multivariable models showed that immunoglobulin and/or plasma exchange were significant predictors of recovery. Employment and recovery factors (positive interactions, work support and changes at work or home, physical activity and therapy), though associated with recovery, did not reach statistical significance.

**Conclusion:**

The questionnaire demonstrated good internal reliability of scales and subscales and construct validity for people following GBS.

**Patient Contribution:**

Patients were involved in developing and piloting the questionnaire.

## 1 INTRODUCTION

Guillain‐Barré syndrome (GBS) is a rare inflammatory disorder, affecting peripheral nerves, with an incidence of 1–2/100,000 per year.^1^ The disorder produces symmetrical weakness and numbness of the limbs, progressing proximally usually over 2–4 weeks, with symptom onset to nadir within 6 weeks. There are several atypical variants of GBS including Miller Fisher syndrome, which can affect cranial nerves (causing eye, facial or swallowing problems), balance and coordination and Bickerstaff brainstem encephalitis, which also affects the central nervous system, and although some investigations, such as nerve conduction studies and cerebrospinal fluid analysis are supportive, the diagnosis is largely made clinically.^2^

The severity of GBS is variable, with patients with mild GBS experiencing little disability and recovering spontaneously, but in 20%–30% of cases, a more severe generalized form rapidly progresses to affect facial and respiratory muscles, and causes symptoms leading to more severe disability or even death.^3^ Treatment, particularly for more severe cases, may involve life‐saving supportive therapy at the intensive care unit, administration of intravenous immunoglobulins or plasma exchange (PE), which significantly shortens the time to (but not the extent of) recovery,^4^, ^5^ followed by rehabilitation.^6^

Large prospective studies such as the International GBS Outcome Study (IGOS) have shown wide variations in outcomes.^7^, ^8^ Many patients, particularly those with mild forms of GBS, recover completely within 1–2 years, but others will have residual or long‐lasting physical, psychological or social sequelae. Physical effects include pain, chronic fatigue and difficulty in walking.^3^, ^9^ Reported psychological symptoms include experiences of sleep disturbance, anxiety or posttraumatic stress disorder, which can affect a person's daily life activities, work or social function over years.^10^

A recent systematic review and metasynthesis of qualitative studies of people with GBS showed the complexity of experience of the illness, its care and rehabilitation from illness onset to hospitalisation with acute symptoms, recovery and adjustment in the case of longer‐term problems.^11^ This and other studies of patient‐reported experiences of neurological conditions have identified common factors associated with care quality.^12^ A further qualitative study exploring those factors associated with recovery demonstrated the importance of early diagnosis, positive experiences of inpatient care, active support for recovery and good communication and information provision.^13^

Quality in healthcare is widely considered to consist of three interrelated components: safety, effectiveness and experience.^14^, ^15^ Tools such as the Inflammatory Rasch‐built Overall Disability Scale, the Medical Research Council sum score and the Inflammatory Neuropathy Cause and Treatment disability score have been used to monitor the effectiveness of treatment and disease progression.^16^ Healthcare experiences include ‘experiences of what health services and staff are like and do’ and experiences of how they feel services ‘enable [them] to be and do what [they] value being and doing within and beyond [their] healthcare encounters’.^17^ Patient‐reported experience measures (PREMs) are widely used to assess patient experience as a key aspect of quality.^18^, ^19^ This is also relevant and important for patients' experiences of conditions such as GBS and its variants.

We aimed to develop and validate a questionnaire to quantify experiences of people with GBS.

## 2 METHODS

### 2.1 Design

We used a cross‐sectional design using a self‐administered online questionnaire survey designed to explore symptoms, care experiences and recovery in people who previously had GBS.

### 2.2 Questionnaire development

Questionnaire domains and items were based on a systematic review and metasynthesis of qualitative studies^11^ and an interview study of people with the condition.^13^ The domains for people who previously had GBS included participant characteristics; severity of symptoms (physical, psychological and social) at each stage of illness; medical health‐seeking experience; treatment and care experiences; follow‐up and support; and social or work‐related experience.

The initial questionnaire was piloted with four people who had recovered from GBS, of whom two had taken part in an earlier interview study.^13^ The questionnaire was also discussed with the Guillain‐Barré Syndrome and Associated Inflammatory Neuropathies (GAIN) charity, the Healthier Ageing Patient and Public Involvement group at the University of Lincoln and members of the research team. Comments and suggestions were used to revise some of the questions to ensure that they were appropriate for the intended population of GBS patients.

### 2.3 Participant recruitment and data collection

Ethical approval was obtained from the University of Lincoln Human Ethics Committee (2019‐Jul‐0738). A convenience sample of people with GBS living in the United Kingdom was recruited through a social media advertisement posted on Twitter and the UK GBS charity, GAIN website, Facebook page and member list. Information about the research (including consent and a link to the questionnaire) was posted at the GAIN and University of Lincoln Community and Health Research Unit websites (https://www.cahru.org.uk/) accessible to potential participants. Participants, who self‐identified with a diagnosis of GBS, consented and completed the questionnaire online. No financial incentives were given to responders. Participants were encouraged to contact a member of the research team (J. A.) if they needed further information or assistance to complete the questionnaire. The survey remained open for 2 months (August and September 2019), and once completed by participants, the questionnaire was retrieved and stored securely for analysis.

### 2.4 Data analysis

The internal consistency of the GBS questionnaire was assessed using Cronbach's *α*.^20^ This test was used to establish the level of agreement between items belonging to the same scale. Four main scales were developed, which contained items scored on a 7‐point Likert scale including *symptoms*, *care received*, *factors affecting recovery* and *information provided*. Some of these scales were divided into further subscales: initial, in‐hospital, after‐hospital and current symptoms as well as care received in hospital and after discharge from hospital.

Factor analyses were run to identify questionnaire subscales. The scales included were suitable for this type of analysis as indicated by the Kaiser–Meyer–Olkin measure, which was higher than 0.7 for all of them. Retained factors were those with eigenvalues greater than 1 and items with loadings higher than 0.4.^21^ As such, the scales included in the factor analysis (FA) were symptoms (initial, in hospital, residual and current), factors affecting recovery and information provided.

Multivariate linear regression models were used to identify the factors predicting recovery. Two regression models were run: The first one using the scales of the questionnaire as predictors and the second one using the subscales derived from factor analyses as the main predictors together with demographic characteristics that might have influenced the outcome. These demographic predictors included age and the binary variables: sex (female or male), employment status (employed or unemployed) and living with someone else or alone. The recovery score, which was used as the main outcome, was computed using the formula: r*ecovery score* = *mean score of in hospital symptoms − mean score of present symptoms*.

The assumption of normality was met as indicated by both histograms and P–P plots of residuals. Homoscedasticity was present as indicated by scatterplots. The assumption of no multicollinearity was also met for both models as indicated by Durbin Watson tests with values close to 2 (1.93 for the first model and 2.12 for the second model), tolerance values higher than 1 and Variance Inflation Factor values smaller than 10.

## 3 RESULTS

### 3.1 Responder characteristics

In total, 291 participants responded fully or partially to the questionnaire. Table 1 shows the demographic characteristics of the participants who responded. Of the responders, 123 (45.6%) were aged between 60 and 79 years; 140 (51.9%) were male and 130 (48.1.%) were female. Most participants were of White ethnicity (264, 97.8%) compared with the minority, who were either BAME (2, 0.7%) or mixed race (3, 1.1%) or other (1, 0.4%). 178 (65.9) were married or in civil partnership compared with 57 (21.1%) who were single or 35 (13.0%) who did not declare their marital status. At the time of the survey, most participants (252, 86.6%) resided in the United Kingdom compared with non‐UK residence (39, 13.4%). More participants were retired from work (89, 38%) compared with those in full‐time employment (55, 23.5%) or part‐time work (31, 13.2%) or those on disability and/or other benefits and not working (30, 12.8%). At the time of the study, the majority of participants (177, 65.6%) were living with their spouse compared with other family members (27, 10%) or alone (38, 14.1%) or other (27, 10%).

**Table 1:** Participants' demographic characteristics

Characteristic		Number (*N*)	Percentage (%)
Age (years)			
Below 18		2	0.7
19–39		40	14.8
40–59		96	35.6
60–79		123	45.6
80+		9	3.3
Total		270	100
Sex			
Female		130	48.1
Male		140	51.9
Total		270	100
Ethnicity			
White		264	97.8
BAME		2	0.7
Mixed race		3	1.1
Other		1	0.4
Total		270	100
Marital status			
Married		168	62.2
Civil partnership		10	3.7
Single		57	21.1
Other		34	12.6
Prefer not to say		1	0.4
Total		270	100
Residence			
United Kingdom		252	86.6
Non‐United Kingdom		39	13.4
Total		291	100
Employment status			
In full‐time time work		55	23.5
In part‐time work		31	13.2
In work with disability and/or other benefits		6	2.6
On disability and/or other benefits and not working		30	12.8
Unemployed		3	1.3
Retired		89	38
Other		20	8.5
Total		234	100
Household status			
Spouse		177	65.6
Other family member		27	10
Alone		38	14.1
Other		27	10
Prefer not to say		1	0.4
Total		270	100

Disease characteristics of the responders are shown in Table 2. Most responders had a diagnosis of GBS (202, 74.8%) compared with chronic inflammatory demyelinating polyradiculoneuropathy (CIDP) (46, 17.0%) or a related condition (22, 8.1%). Most responders sought help within the first 3 days (158, 60.3%) of feeling unwell and help was most commonly sought from a general practitioner (GP) surgery (163, 62.2%) compared with the emergency department (67, 25.6%). Most responders (166, 64.6%) received a GBS diagnosis or its variant rather than a different diagnosis (91, 35.4%), 106 responders receiving it on their first visit.

**Table 2:** Participants' disease characteristics

Characteristic		Number (*N*)	Percentage (%)
Diagnosis			
GBS		202	74.8
CIDP		46	17.0
Related condition		22	8.1
Total		270	100
Help sought after (days)			
1–3		158	60.3
4–6		40	15.3
7–9		19	7.3
10–14		14	5.3
15–28		11	4.2
>28		20	7.6
Total		262	100
Help sought from			
General practitioner surgery		163	62.2
Emergency department		67	25.6
Other (please state)		32	12.2
Total		262	100.0
Delay in days after first visit			
1–7 days		161	61.7
8–14 days		31	11.9
15–28 days		28	10.7
More than 4 weeks (please state how many weeks approximately)		41	15.7
Total			
Number of consultations before diagnosis			
1		106	44.9
2		42	17.8
3		38	16.1
4		18	7.6
5		13	5.5
≥6		5	7.8
Too many to recall		6	2.5
Total		236	100.0
Another/other diagnosis			
No		166	64.6
Yes		91	35.4
Place treatment			
Intensive care unit (ICU)		40	16.7
Hospital general ward		36	15.1
Received			
Hospital general ward and ICU		76	31.8
Hospital general ward, outpatient		16	6.7
Hospital general ward, Regional Neurological centre		71	29.7
Total		239	100
Year of diagnosis			
Before 2000		49	18.1
2000–2009		51	19.9
2010–2019		170	63.0
Total		270	100.0

Abbreviations: CIDP, chronic inflammatory demyelinating polyradiculoneuropathy; GBS, Guillain‐Barré syndrome.

Overall, 116 (of 291 responders, i.e., 43%) were diagnosed between 2015 and 2019, and time to diagnosis was usually 1–7 days (161, 61.7%) compared with later (100, 38.3%). Responders were generally treated in a hospital general ward or an intensive care unit (76, 31.8%) and a hospital general ward or a regional neurological unit (71, 29.7%).

### 3.2 Reliability of scales and subscales

The reliability (internal consistency) of the main scales was excellent for *symptoms* (*α* > .9), good for i*nformation provided* and for f*actors affecting recovery* (*α* > .8) and acceptable for c*are received* (*α* ≥ .7). Importantly, none of the scales had poor reliability (*α* ≤ .6).^20^ Overall, these results presented in Table 3 indicate that the questionnaire was a reliable measure, with good internal consistency.

**Table 3:** Reliability of each scale and subscale of the GBS questionnaire

	Cronbach's *α*	Items	Observations
Scales			
Symptoms	0.94	56	60
Care received	0.70	9	175
Factors affecting recovery	0.80	28	47
Information provided	0.88	11	208
Subscales			
Symptoms			
Initial symptoms	0.89	13	158
Hospital symptoms	0.88	13	156
Residual symptoms	0.86	13	163
Current symptoms	0.89	13	172
Care received			
In hospital	0.65	4	232
After	0.56	4	181

Abbreviation: GBS, Guillain‐Barré syndrome.

Further subscales were identified following FA. Symptoms included the following subscales: peripheral nerve symptoms, cranial nerve and respiratory symptoms and psychological symptoms. Factors affecting recovery included positive interactions, work support, changes at work, changes at home, physical activity, therapy and other subscales. Because the subscale, ‘other’, had a very low reliability, the items of this subscale were introduced separately in the regression models. These two items were immunoglobulin treatment and caring responsibilities. The information provided was divided into two further subscales: provided by specialists including physiotherapists, occupational therapists and by nonspecialists including nurses, junior doctors and GPs. A detailed account of each subscale and the items in these can be seen in Table S1. The internal reliability of the new subscales is presented in Table 4.

**Table 4:** Internal consistency of subscales identified following factor analysis

	Cronbach's *α*	Items	Observations
Subscales			
Prompted symptoms			
Peripheral nerve	0.85	7	174
Cranial nerve/respiratory	0.86	3	178
Psychological	0.78	3	180
Hospital symptoms			
Peripheral nerve	0.86	7	187
Cranial nerve/respiratory	0.84	3	181
Psychological	0.75	3	182
Residual symptoms			
Peripheral nerve	0.85	7	187
Cranial nerve/respiratory	0.59	3	179
Psychological	0.79	3	185
Current symptoms			
Peripheral nerve	0.91	6	194
Cranial nerve/respiratory	0.67	3	191
Psychological	0.78	4	185
Factors affecting recovery			
Positive interactions	0.84	6	213
Work support	0.85	4	191
Changes at work	0.65	5	184
Changes at home	0.66	4	202
Physical activity	0.59	3	210
Therapy	0.52	2	209
Other	0.20	3	190
Information provided			
Nonprofessionals	0.72	4	219
Professionals	0.67	3	223

**Figure 1:**
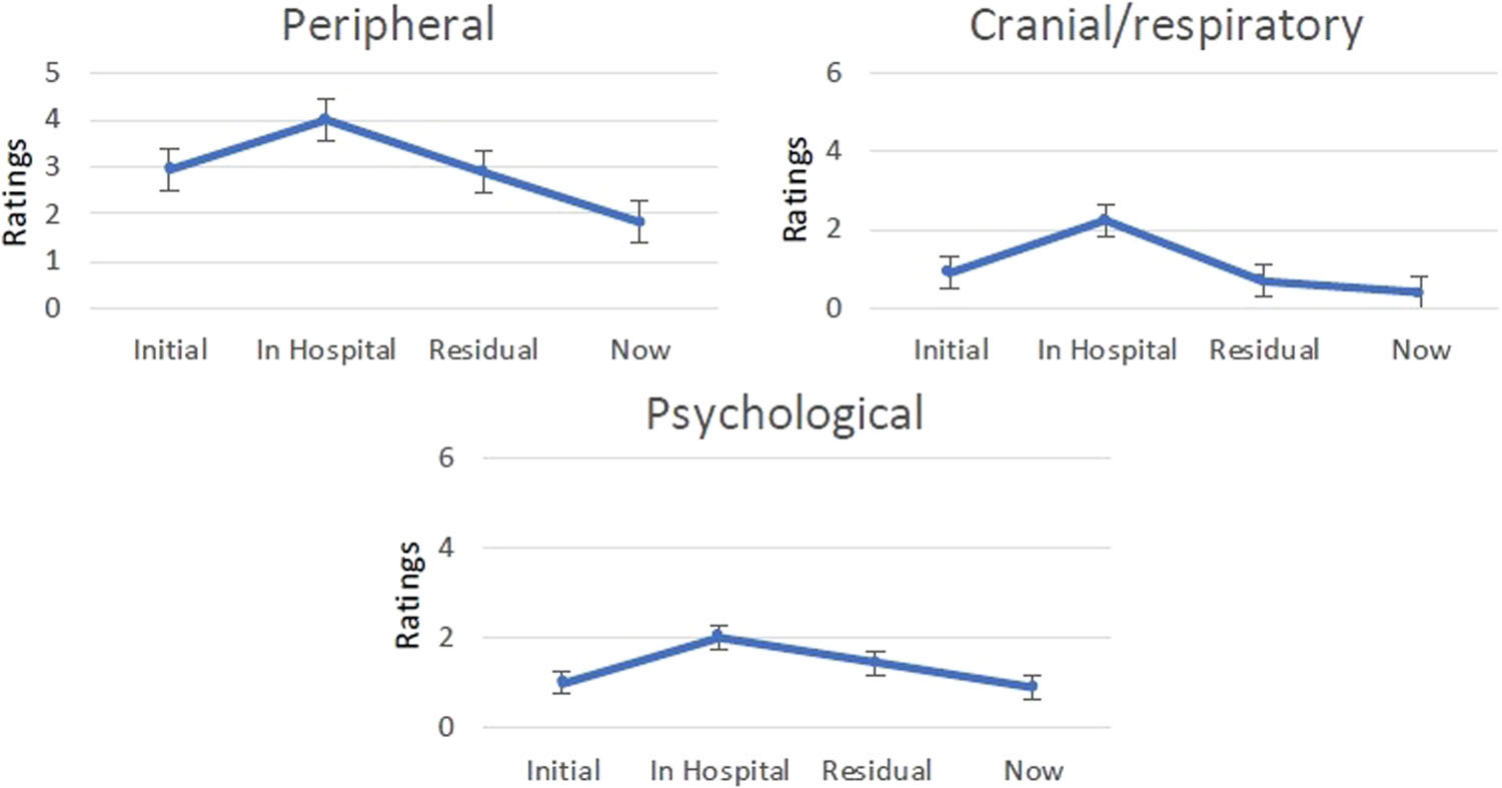
Severity of symptoms for each subscale over time

The reported severity of symptoms for each subscale at different time points (initial before admission to hospital, in hospital, residual and current, i.e., when responders were completing the questionnaire) indicated that symptoms were most severe when responders were in hospital and those affecting the peripheral nervous system were most prominent (Figure 1).

**Figure 2:**
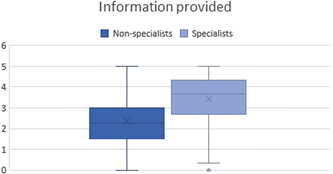
Boxplot showing satisfaction with information provided by specialists compared with nonspecialists

Responders were more satisfied with the information provided by specialists rather than nonspecialists (Figure 2 and Table S2).

**Figure 3:**
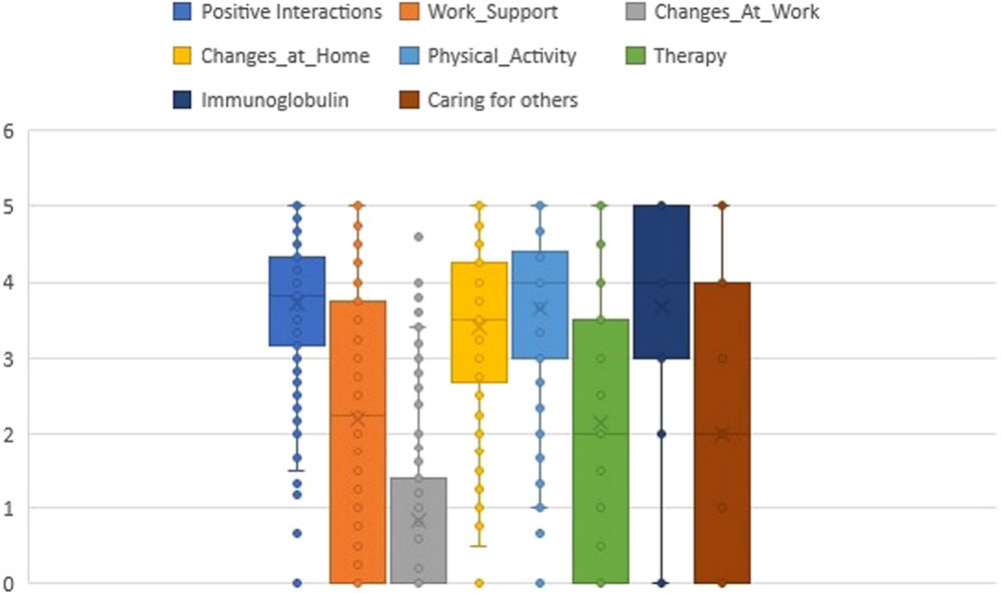
Boxplot of the main factors affecting recovery illustrating the distribution of average scores for each factor, three representing the median

A combination of physical, psychological and social factors was associated with recovery; these factors were identified following the FA and an average score was calculated for each factor; details can be seen in Table S3. The factors considered by responders to be most important for recovery were positive social interactions, physical activity including physiotherapy and movement, changes made at home and immunoglobulin treatment (Figure 3).

### 3.3 Prediction models

Multivariable regression models were fitted to the data. The predictors used were gender, age, employment status and living alone or with someone, since these variables had been previously shown to influence recovery.^11^, ^13^ In the first regression model, the main scales of the questionnaire (shown in Table 5) were included as predictors. In the second regression model, the main subscales of the questionnaire (shown in Table 6) were included in the model. The results indicated that immunoglobulin and/or PE treatment were significant predictors of recovery.

**Table 5:** Questionnaire scales predicting the recovery score

Predictors	Recovery score			
	*B*	*β*	95% CI of *B*	*p*‐Value
Gender	−0.33	−.31	−0.83, 0.20	.23
Age	−0.29	−.15	−0.71, 0.14	.19
Employment	0.50	.20	−0.07, 1.07	.09
Household status	0.49	.16	−0.19, 1.18	.16
Care received	0.04	.03	−0.37, 0.44	.86
Factors affecting recovery	0.36	.24	−0.03, 0.74	.07
Information provided	0.22	.20	−0.10, 0.54	.17
*R* ^2^ = .32, *F* (7,64) = 4.24, *p* = .001				

Abbreviation: CI, confidence interval.

**Table 6:** Questionnaire subscales predicting the recovery score

Predictors	Recovery score			
	*B*	*β*	95% CI of *B*	*p*‐Value
Gender	0.18	.09	−0.73, 1.08	.69
Age	−0.30	−.19	−0.94, 0.33	.33
Employment	0.57	.27	−0.51, 1.65	.29
Household status	−0.35	−.12	−1.54, 0.84	.54
Care received in hospital	−0.18	−.17	−0.83, 0.47	.57
Care received after	−0.09	−.10	−0.48, 0.31	.66
Information provided by nonspecialists	0.09	.12	−0.26, 0.44	.59
Information provided by specialists	0.24	.29	−0.25, 0.73	.33
Positive social interactions	0.36	.37	−0.21, 0.92	.21
Work support	−0.06	−.11	−0.33, 0.21	.67
Changes at work	−0.04	−.06	−0.35, 0.26	.78
Changes at home	0.04	.05	−0.42, 0.51	.85
Physical activity	−0.21	−.26	−0.73, 0.30	.40
Therapy	0.10	.16	−0.17, 0.37	.44
Immunoglobulin and/or plasmapheresis	0.23	.38	0.01, 0.45	.04*
Caring responsibilities	0.01	.02	−0.21, 0.24	.91
*R* ^2^ = .55, *F*(16,22) = 1.68, *p* = .13				

Abbreviation: CI, confidence interval.

* *p* < .05.

## 4 DISCUSSION

### 4.1 Main findings

The high completion rate and low rates of missing data for most questions supported the content and face validity of the questionnaire. The questionnaire showed reliability as excellent for *symptoms*, good for i*nformation provided* and f*actors affecting recovery* and acceptable for c*are received* and symptom subscales. Physical, psychological and social factors were associated with recovery, and concordance with recent studies^11^, ^13^ supports construct validity. Factors considered by responders to be most important for recovery were positive social interactions, physical activity, changes made at home and immunoglobulin treatment. Responders were more satisfied with information provided by specialists rather than nonspecialists. Multivariable models showed that immunoglobulin and/or PE treatment were significant predictors of recovery.^4^ Being in employment and recovery factors in combination (positive social interactions, support and changes at work support, changes at home, physical activity and counselling or occupation therapy) were positively associated with recovery, but this did not reach statistical significance.

### 4.2 Comparison with the existing literature

Although many people with GBS are told that they will recover and some do so completely, many are still affected in the longer term. Early results from the largest ongoing prospective study, the IGOS,^7^ have shown that 8% could not walk and 7% had died at 1 year, with wide international variations in outcome.^8^ Previous studies have also shown long‐term neurological deficits in most patients after a year or beyond.^22^, ^23^ Furthermore, a third had changed work or were affected in their functional ability and half had altered their leisure activities.^22^ Psychological^24^ and social dysfunction^25^ often persist longer term, affecting health‐related quality of life.^26^

Previous research has suggested a wide variation in positive and negative experiences at various stages of treatment and recovery from GBS.^11^, ^13^ We also found wide variations in experiences of care from different healthcare professionals during the illness journey, with the most positive experiences of care in hospital, from consultants, followed by nurses and therapists. Consultants, followed by physiotherapists were also rated highly for care at follow‐up, and although in this study physical, psychological and social support were (nonsignificantly) associated with improvement in symptoms, experiences of care and psychosocial support remain important aspects of quality of care.

Rehabilitation studies, involving careful follow‐up, show positive benefits of rehabilitation on function^27^ and mortality^28^ before discharge from hospital, but intensive physiotherapy beyond 6 months was also found to improve functional outcomes.^6^ Responders in our study valued physiotherapy and perceived this to improve their recovery, but shortfalls in provision for both inpatient and outpatient rehabilitation have been found in previous studies.^29^, ^30^

Positive social interactions and changes at home were also associated with recovery in this study. Positive social interactions include family or peer support.^13^ A systematic review found that peer support as a potential intervention for recovery in critical care populations reduced psychologic morbidity and improved self‐efficacy, although the quality of included studies was low.^31^ Finally, complementary therapies such as acupuncture, vitamins and hyperbaric oxygen have been used as an adjunct to conventional treatment, but the only nonrandomized study was deemed of low quality.^32^

### 4.3 Strengths and limitations

The number of questionnaires returned was sufficient for the planned analysis, and most participants who began completed the questionnaire. The sample was not intended to be representative of GBS patients as our main aim was to explore the reliability and validity of the questionnaire for measuring responders' experience of GBS and its care. As such, the sample comprised mostly participants over 40 years old, while patients with more severe sequelae were less likely to respond. The diagnosis of GBS or a variant was based on responder self‐identification and we were unable to confirm this from medical records. However, we considered it unlikely that people with conditions other than GBS would identify themselves as having GBS and then go on to complete an extensive questionnaire of their experiences. Some participant characteristics such as year of and time since diagnosis, time to seek help, number of consultations and delay before diagnosis, place of treatment and length of hospital stay may have been subject to recall bias.

### 4.4 Implications for practice and research

The responses to the survey confirmed recent studies suggesting that various physical, psychological and social factors were associated with recovery.^11^, ^13^ Because the survey showed good evidence of face and construct validity and internal consistency, it could be used to assess patient experience and how experience of care and support could be improved in a larger population of people with GBS.

Further research needs to be done to develop patient‐reported outcome measures^33^ and PREMs for GBS beyond traditional disability measures such as the GBS Disability Scale.^34^ The experience scales developed in this survey could be used to develop and evaluate the effect of interventions designed to improve experiences at various stages of treatment and recovery including in the longer term, including better access to rehabilitation and innovative social interventions such as peer or employer support.

## 5 CONCLUSION

Our findings showed that the GBS patient experience survey showed characteristics of a good measure, with evidence of internal consistency and construct validity. The GBS patient experience questionnaire should be tested more widely to seek further evidence of reliability, construct validity and sensitivity to differences in care and setting.

## CONFLICT OF INTERESTS

The authors declare that there are no conflict of interests.

## AUTHOR CONTRIBUTIONS

Aloysius N. Siriwardena had the original idea for the study. The study was designed by Aloysius N. Siriwardena and Joseph N. A. Akanuwe, supported by Zahid B. Asghar, Vanessa Botan, Despina Laparidou, Ffion Curtis, Jennifer Jackson and Timothy L. Hodgson. Fieldwork was conducted by Joseph N. A. Akanuwe and analysis by Vanessa Botan, supported by Aloysius N. Siriwardena, Zahid B. Asghar, Despina Laparidou, Ffion Curtis and Jennifer Jackson. Aloysius N. Siriwardena wrote the first draft of the paper and all the authors edited and approved the paper.

## ETHICS STATEMENT

This study was approved by the Lincoln University Ethics Committee. All interviewees provided informed consent to participate. This study was performed in accordance with the Declaration of Helsinki.

## Supporting information

Supporting information.

## Data Availability

The data analysed during this study are available from the corresponding author on reasonable request.
