# Supplementary material for: Patient‐reported symptoms and experience following Guillain‐Barré syndrome and related conditions: Questionnaire development and validation
Source: Health Expect. 2021 Oct 1;25(1):223–31. doi: 10.1111/hex.13367 (PMC8849366; doi:10.1111/hex.13367)
Supplement: Supplementary file 1 — Supporting information. [file HEX-25-223-s001.docx]

**SUPPLEMENTARY TABLES**

**Table S1 Rating of experience and factors affecting recovery**

| **Experience of care or recovery** | **Missing**  **N (%)** | **Negative**  **N (%)** | **Neutral**  **N (%)** | **Positive**  **N (%)** | **Total**  **N (%)** |
| --- | --- | --- | --- | --- | --- |
| **How would you rate your experience of care from the following?** |  |  |  |  |  |
| Hospital doctors/consultants | 0 (0) | 17 (7.1) | 29 (12.1) | 193 (80.8) | 239 (100) |
| Hospital nursing care | 4 (1.7) | 18 (7.6) | 39 (16.5) | 176 (74.3) | 237 (100) |
| Hospital physiotherapy | 18 (7.5) | 30 (12.6) | 25 (10.5) | 166 (69.5) | 239 (100) |
| Hospital occupational therapy | 48 (20.5) | 23 (9.8) | 32 (13.7) | 131 (56.0) | 234 (100) |
| **How would you rate your experience of follow-up treatment following?** |  |  |  |  |  |
| General Practitioner | 32 (14.7) | 39 (17.9) | 46 (21.1) | 101 (46.3) | 218 (100) |
| Consultant | 18 (8) | 26 (11.6) | 42 (18.8) | 138 (61.6) | 224 (100) |
| Pain clinic | 156 (80.4) | 13 (6.7) | 7 (3.6) | 18 (9.3) | 194 (100) |
| Physiotherapy | 32 (14.2) | 25 (11.1) | 33 (14.6) | 136 (60.2) | 226 (100) |
| Occupational therapy | 91 (43.8) | 23 (11.1) | 20 (9.6) | 74 (35.6) | 208 (100) |
| **Which of the following affected your recovery?** |  |  |  |  |  |
| Treatment with Ig and/or plasma exchange | 36 (15.5) | 6 (2.6) | 23 (9.9) | 167 (72.0) | 232 (100) |
| Counselling or other psychological treatment | 125 (58.7) | 17 (8) | 25 (11.7) | 46 (21.6) | 213 (100) |
| Physiotherapy | 25 (10.8) | 6 (2.6) | 13 (5.6) | 187 (81.0) | 231 (100) |
| Occupational therapy | 64 (29) | 6 (2.7) | 23 (10.4) | 128 (57.9) | 221 (100) |
| Exercise/activity | 15 (6.6) | 8 (3.5) | 15 (6.6) | 188 (83.2) | 226 (100) |
| Self-management | 10 (4.5) | 3 (1.4) | 10 (4.5) | 197 (89.5) | 220 (100) |
| Modifications to home | 85 (39.5) | 6 (2.8) | 11 (5.1) | 113 (52.6) | 215 (100) |
| Moving from hospital to home | 25 (11.3) | 18 (8.1) | 19 (8.6) | 159 (71.9) | 221 (100) |
| Adaptation to the condition | 22 (10) | 30 (13.6) | 23 (10.5) | 145 (65.9) | 220 (100) |
| Staying positive about the future | 4 (1.7) | 19 (8.2) | 16 (6.9) | 193 (83.2) | 232 (100) |
| Seeing current situation as an opportunity for change | 27 (12.5) | 37 (17.1) | 48 (22.2) | 104 (48.1) | 216 (100) |
| Responsibilities caring e.g. for spouse, children or an older parent | 92 (42.4) | 28 (12.9) | 31 (14.3) | 66 (30.4) | 217 (100) |
| Relationships with others | 18 (8.1) | 39 (17.6) | 31 (14) | 134 (60.4) | 222 (100) |
| Support from family members | 6 (2.6) | 11 (4.7) | 10 (4.3) | 205 (88.4) | 232 (100) |
| Support from friends | 6 (2.6) | 16 (6.9) | 20 (8.7) | 189 (81.8) | 231 (100) |
| Engaging in social activities | 17 (7.7) | 42 (18.9) | 31 (14) | 132 (59.5) | 222 (100) |
| Others knowing or becoming aware you were ill or disabled | 9 (4.1) | 34 (15.3) | 52 (23.4) | 127 (57.2) | 222 (100) |
| Other | 55 (68.8) | 7 (8.8) | 1 (1.3) | 17 (21.3) | 80 (100) |
| **Which of the following work-related factors affected your recovery?** |  |  |  |  |  |
| Work related benefits | 106 (54.1) | 10 (5.1) | 18 (9.2) | 62 (31.6) | 196 (100) |
| Phased return to usual work | 96 (49) | 9 (4.6) | 5 (2.6) | 86 (43.9) | 196 (100) |
| Change to part-time | 147 (77.8) | 2 (1.1) | 7 (3.7) | 33 (17.5) | 189 (100) |
| Change to job role or duties | 137 (71.7) | 3 (1.6) | 7 (3.7) | 44 (23.0) | 191 (100) |
| Change of job | 157 (83.1) | 3 (1.6) | 2 (1.1) | 27 (14.3) | 189 (100) |
| Adaptations at work) | 146 (77.2) | 5 (2.6) | 9 (4.8) | 29 (15.3) | 189 (100) |
| Support from your employer | 84 (43.5) | 16 (8.3) | 7 (3.6) | 86 (44.6) | 193 (100) |
| Support from work colleagues | 79 (40.5) | 12 (6.2) | 10 (5.1) | 94 (48.2) | 195 (100) |
| Early retirement | 151 (77.4) | 3 (1.5) | 8 (4.1) | 33 (16.9) | 195 (100) |
| Other | 99 (92.5) | 4 (3.7) | 2 (1.9) | 2 (1.9) | 107 (100) |

**Table S2 Rating of information provided**

| **Variable** | Missing  N (%) | Negative  N (%) | Neutral  N (%) | Positive  N (%) | Total  N (%) |
| --- | --- | --- | --- | --- | --- |
| **How would you rate the information provided?** |  |  |  |  |  |
| GP | 29 (12.5) | 104 (44.8) | 39 (16.8) | 60 (25.9) | 232 (100) |
| Practice Nurse | 81 (35.7) | 77 (33.9) | 34 (15) | 35 (15.4) | 227 (100) |
| Junior Doc | 23 (10.2) | 65 (28.9) | 61 (27.1) | 76 (33.8) | 225 (100) |
| -Hospital Nurse) | 15 (6.7) | 54 (24) | 58 (25.8) | 98 (43.6) | 225 (100) |
| Consultant | 4 (1.7) | 22 (9.5) | 25 (10.8) | 181 (78.0) | 232 (100) |
| Physiotherapist | 21 (9.1) | 30 (12.9) | 26 (11.2) | 155 (66.8) | 232 (100) |
| Occupational therapist(s) | 58 (25.9) | 23 (10.3) | 37 (16.5) | 106 (47.3) | 224 (100) |
| at the time around diagnosis | 0 (0) | 66 (28.3) | 68 (29.2) | 99 (42.5) | 233 (100) |
| during your hospital stay) | 0 (0) | 50 (21.7) | 61 (26.5) | 119 (51.7) | 230 (100) |
| at the time discharged from hospital | 0 (0) | 59 (25.8) | 65 (28.4) | 105 (45.9) | 229 (100) |
| After you left hospital | 0 (0) | 76 (33.8) | 71 (31.6) | 78 (34.7) | 225 (100) |
| Health staff | 8 (3.6) | 58 (25.9) | 67 (29.9) | 91 (40.6) | 224 (100) |
| Charities |  | 9 (4) | 17 (7.5) | 177 (78.0) | 227 (100) |
| Social media | 76 (35.3) | 7 (3.3) | 41 (19.1) | 91 (42.3) | 215 (100) |
| Online | 77 (51.3) | 3 (2) | 22 (14.7) | 48 (32) | 150 (100) |
| Others | 93 (85.3) | 1 (0.9) | 4 (3.7) | 11 (10.1) | 109 (100) |

**Table S3 Items loading on each of the subscales of the questionnaire**

| **Sub-scales** | **Items** |
| --- | --- |
| **Symptoms** |  |
| Peripheral nerve | Reduced mobility |
| symptoms | Numbness |
|  | Weakness |
|  | Paralysis |
|  | Tiredness or fatigue |
|  | Tingling or ‘pins and needles’ sensations |
|  | Pain |
| Cranial nerve/respiratory | Difficulty speaking |
| symptoms | Problems with eyesight or vision |
|  | Difficulty breathing |
| Psychological symptoms | Sleep difficulties |
|  | Mental health problems (e.g. anxiety, depression, etc.) |
|  | Difficulty with memory or thinking |
| **Factors affecting recovery** |  |
| Positive social interactions | Support from friends |
|  | Others knowing or becoming aware you were ill or disabled |
|  | Engaging in social activities |
|  | Relationships with others |
|  | Support from family members |
|  | Staying positive about the future |
| Work support | Support from your employer |
|  | Support from your work colleagues |
|  | Phased return to usual work |
|  | Work related benefits |
| Changes at work | Change to job role or duties |
|  | Change of job |
|  | Adaptations at work |
|  | Change to part-time |
| Changes at home | Modifications to home |
|  | Moving from hospital to home |
|  | Adaptation to the condition |
|  | Self-management |
| Physical activity | Exercise/ activity |
|  | Physiotherapy |
| Therapy | Counselling or other psychological treatment |
|  | Occupational therapy |
| Other | Responsibilities caring (e.g. for spouse, children, or an older parent) |
|  | Treatment with immunoglobulins and/or plasma exchange |
| **Information provided** |  |
| Specialists | Physiotherapists |
|  | Occupational therapists |
|  | Consultant |
| Non-specialists | Hospital nurses |
|  | General practitioners (GPs) |
|  | Practice nurses |
|  | Junior hospital doctors |
